# The ephrin receptor EphB2 regulates the connectivity and activity of enteric neurons

**DOI:** 10.1016/j.jbc.2021.101300

**Published:** 2021-10-11

**Authors:** Raphael Bodin, Vincent Paillé, Thibauld Oullier, Tony Durand, Philippe Aubert, Catherine Le Berre-Scoul, Philippe Hulin, Michel Neunlist, Moustapha Cissé

**Affiliations:** 1 Inserm, TENS, The Enteric Nervous System in Gut and Brain Diseases, IMAD, Université de Nantes, Nantes, France; 2 UMR 1280 Physiologie des Adaptations Nutritionnelles, INRA, Institut des Maladies de l'Appareil Digestif, Université de Nantes, Nantes, France; 3 Plateforme MicroPICell, SFR Santé, Nantes, France

**Keywords:** EphB2, synaptic-associated proteins, enteric neurons, enteric glial cells, connectivity, ephrinB2, enteric nervous system, synapsin I, PSD95, CNS, central nervous system, DMEM, Dulbecco's modified Eagle medium, EGC, enteric glial cell, ENS, enteric nervous system, FBS, fetal bovine serum, Fc, fragment crystalizable, mPSC, miniature postsynaptic current, OD, optical density, SAP, synaptic-associated protein

## Abstract

Highly organized circuits of enteric neurons are required for the regulation of gastrointestinal functions, such as peristaltism or migrating motor complex. However, the factors and molecular mechanisms that regulate the connectivity of enteric neurons and their assembly into functional neuronal networks are largely unknown. A better understanding of the mechanisms by which neurotrophic factors regulate this enteric neuron circuitry is paramount to understanding enteric nervous system (ENS) physiology. EphB2, a receptor tyrosine kinase, is essential for neuronal connectivity and plasticity in the brain, but so far its presence and function in the ENS remain largely unexplored. Here we report that EphB2 is expressed preferentially by enteric neurons relative to glial cells throughout the gut in rats. We show that in primary enteric neurons, activation of EphB2 by its natural ligand ephrinB2 engages ERK signaling pathways. Long-term activation with ephrinB2 decreases EphB2 expression and reduces molecular and functional connectivity in enteric neurons without affecting neuronal density, ganglionic fiber bundles, or overall neuronal morphology. This is highlighted by a loss of neuronal plasticity markers such as synapsin I, PSD95, and synaptophysin, and a decrease of spontaneous miniature synaptic currents. Together, these data identify a critical role for EphB2 in the ENS and reveal a unique EphB2-mediated molecular program of synapse regulation in enteric neurons.

Assembly and maintenance of neurons into organized ganglia and the growth, guidance, and connectivity of axons and dendrites are necessary for the establishment of a fine-tuned neural circuitry in the enteric nervous system (ENS). The ENS consists of a complex network of neurons and glia that functions autonomously to provide neural control of major gastrointestinal functions (1). This neuronal network is arranged in two major plexus, *i.e.*, the submucosal and myenteric plexus (2). The early developmental phases of the ENS derived from the neural crest (*i.e.*, migration, proliferation, differentiation) have been extensively studied (1, 3). Nevertheless, mechanisms controlling axon guidance, connectivity of enteric neurons and molecules implicated in these processes remain poorly defined not only during development but also in adulthood. For instance, key proteins referred to as synaptic-associated proteins (SAPs) such as synapsin I, synaptophysin, and PSD95 appear to be crucial determinants for the fine-tuning of synaptic transmission and for synaptic remodeling in the central nervous system (CNS).

SAPs are recognized to play a key role in synaptic mechanisms (*i.e.*, neurotransmitter release, vesicle trafficking) and in the stabilization of synaptic structures in the ENS and the CNS (4, 5, 6). For example, in varicosities of the mouse ENS, PSD95 has been shown to be critical for nitrergic neurotransmission (7). In the CNS, overexpressing PSD95 in cortical neurons reduced the turnover rates of pre- and postsynaptic structures, thus promoting the stabilization of synaptic contacts (8). In addition, in the CNS, a direct interaction between PSD95 and ephrinB3 controls PSD95 localization within synapses and promotes synapse density in cortical neurons (9). Synaptophysin is involved in membrane fusion and neurotransmitter release in the CNS (10, 11). Moreover, increasing synaptophysin expression promotes the formation of neuronal network (12). Synapsins play a major functional role in both the assembly/maintenance of the reserve pool of synaptic vesicles and release during hippocampal neuronal activity (13) and in synapse formation (14, 15). However, their role and the underlying mechanism regulating their expression in the ENS have been less studied.

In this context, the neurotrophic factor EphB2 represents a candidate of choice since it has been largely described as a key regulator of neuronal connectivity and plasticity in hippocampal neurons (16, 17, 18). EphB2 is a member of the eph receptor tyrosine kinase family, members of which are ubiquitously expressed in epithelial and neuronal cells and involved in a variety of developmental processes, neuronal growth, and connectivity/plasticity (19). Fourteen Eph receptors encoded in the human genome are divided into A (EphA1–EphA8 and EphA10) and B (EphB1–EphB4 and EphB6) classes activated by binding to eight different ephrin ligands (9, 20, 21). Eph receptors and ephrins engage in a multitude of activities to mediate contact-dependent communication between cells of the same or different types to control cell morphology, adhesion, movement, proliferation, survival, and differentiation (22, 23, 24). Upon activation, EphB2 receptors could be phosphorylated in its intracellular section, thereby activate tyrosine kinase catalytic activity and engage a cascade of signaling events (25, 26, 27, 28). For instance, ephrinB2-dependant activation of EphB2 induces its tyrosine phosphorylation, leading to an interaction with Kalirin-7 (29) and the Rac1 guanine nucleotide exchange factor in a kinase-dependent manner (27). This interaction promotes a Rac1-dependent actin cytoskeletal remodeling required for dendritic spine morphogenesis in hippocampal neurons (25). Recently, Memic *et al*. (30) identified the expression patterns of ephrin/Eph in the developing mouse and human ENS. Furthermore, a recent study showed that EphB2 was expressed on colonic enteric nerves and could regulate the expression of key molecules involved in synaptic sprouting of myenteric nerves *via* ERK-MAPK and PI3K–protein kinase B pathways in patients with irritable bowel syndrome (31). However, the involvement of additional pathways and the functional impact of EphB2 on enteric neuronal connectivity remain unknown.

Therefore, here we investigate EphB2 spatial localization and signaling in the myenteric plexus of the rat colon and primary cultures of enteric neurons, respectively. Furthermore, we examine its involvement in enteric neuronal connectivity and plasticity through biochemical and electrophysiological approaches.

## Results

### EphB2 expression in different segments of the gut in rat

**Figure 1:**
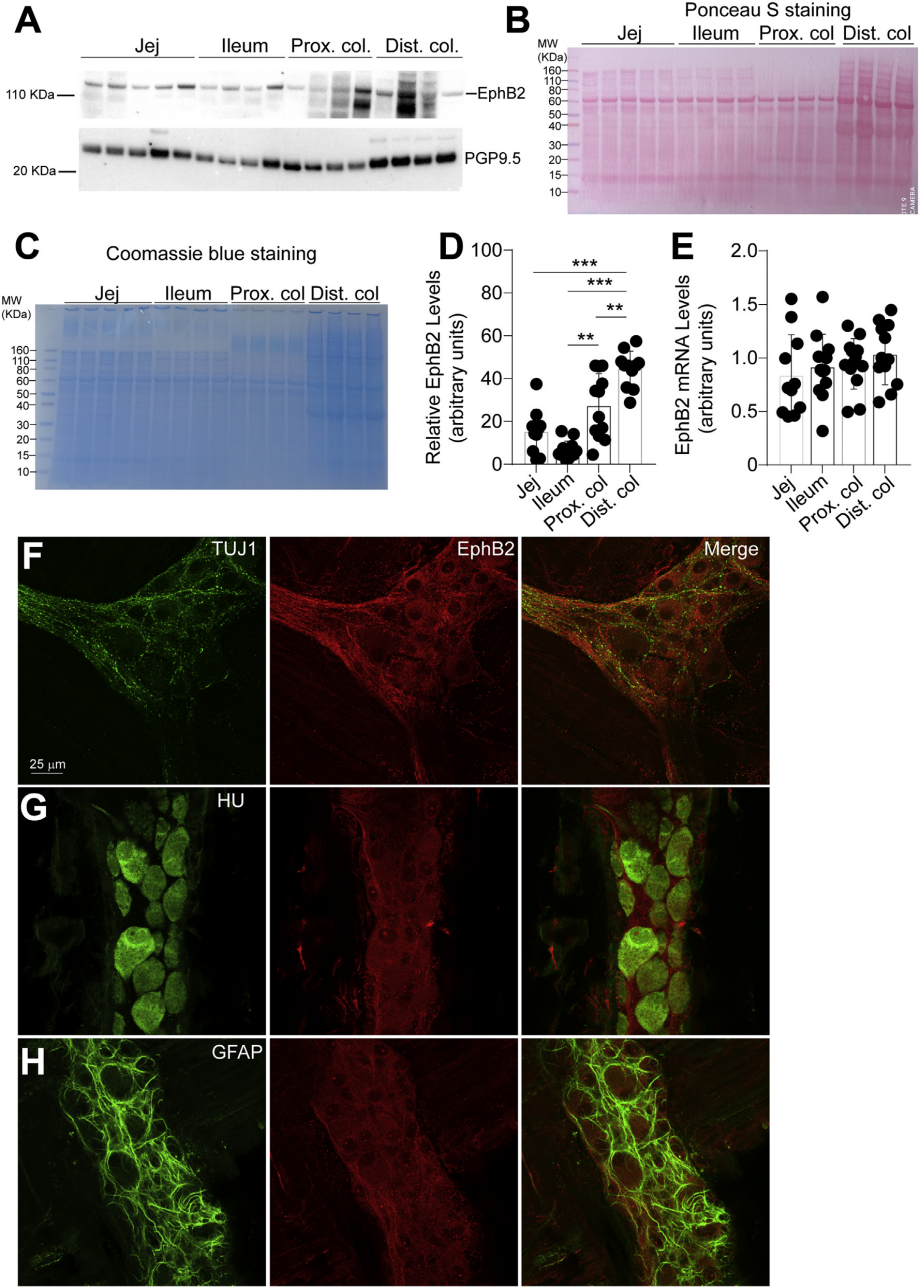
**EphB2 expression in different segments of the rat GI tract.***A*–*C*, EphB2 expression was examined by western blot analysis of full-thickness tissue sections from indicated regions of the rat GI tract. Pgp9.5 was used as a loading control (*A*). Note that pgp9.5 is expressed at higher levels in the distal colon. Furthermore, we observed the apparition of putative EphB2-derived bands in the colon that might be degradation products. Ponceau S (*B*) and Coomassie (*C*) stainings are provided as addition to the traditional loading control. Although equal amount of proteins was loaded for analysis, those stainings show the variability in protein migration profil and relative protein abundance between intestinal segments. Each data point represents the relative adjusted volume of protein band intensity measured by using a rectangle tool of constant volume on each lane of the WB membrane. Data are represented as relative values of protein density in arbitrary units. *D*, densitometric quantitation of western blot signals revealed that EphB2 is present in all segments of the gut (n = 12 sections per segment from 12 rats; jej., jejunum; prox. colon, proximal colon; dist. colon, distal colon; n = 12 sections per segment from 12 animals, F (3, 44) = 18.91, *p* = 0.00000005, ANOVA; Tukey's post-hoc test; ∗∗*p* < 0.001, ∗∗∗*p* < 0.0001). *E*, quantitative RT-PCR analysis of EphB2 mRNA levels in full-thickness tissue sections in the GI tract of adult rats. Note that there is no strict proportionality between EphB2 mRNA levels and translation product levels, likely due to posttranscriptional and posttranslational mechanisms. *F*–*H*, double immunostaining for EphB2 and a specific marker for neural processes βIII tubulin (Tuj1) (*F*), neuronal cell body (Hu) (*G*), or glial marker (GFAP) (*H*) in a whole-mount preparation of myenteric plexus from distal colon of adult rat (6-month-old). Images illustrate that mostly neuronal processes and interganglionic fibers display similar pattern as EphB2 stainings. Images show that pattern of GFAP stainings differs from that of EphB2.

First, we assessed the regional expression of EphB2 in rat small (jejunum and ileum) and large (colon) intestine. Western blot analysis showed that EphB2 is relatively present in all those segments with additional EphB2-derived fragments detectable and neuronal marker PGP9.5 more expressed in the colon (Fig. 1, *A* and *D*). Although the same amount of proteins was loaded for analysis, ponceau S (Fig. 1*B*) and Coomassie blue (Fig. 1*C*) stainings showed a difference in the overall protein profil/content between intestinal segments. Interestingly, we found no difference in levels of EphB2 transcripts between intestinal segments (Fig. 1*E*), suggesting the involvement of a posttranscriptional regulation.

Next, we focused on the cellular expression of EphB2 in the myenteric plexus of the distal colon. Combining confocal imaging with dual labeling with PGP9.5, which stains neuronal soma, processes and interganglionic nerve fiber strands (not shown) or βIII-tubulin (Tuj1) (Fig. 1*F*), which stains neuronal processes, showed a strong localization with neurons, particularly within processes. Further, we observed that in addition to the strong colocalization with Tuj1, a subset of EphB2 resembles/colocalizes with the neuronal cell body marker HuC/D (Fig. 1*G*). In contrast, no localization of EphB2 was observed in enteric glial cells (EGCs) as shown by costainings with the glial marker GFAP (Fig. 1*H*) or S100β (not shown). Interestingly, we found that Kalirin-7, a protein that has been well described as a mediator of EphB2 signaling in the brain (25, 29), was absent in cell body of enteric neurons as shown by colabeling with antibody against HuC/D (Fig. S1*A*). However, we observed that Kalirin-7 was present in neuronal processes as shown by Tuj1 stainings (Fig. S1*B*). This is confirmed by PGP9.5 stainings (Fig. S1*C*). Furthermore, Kalirin-7 was strongly expressed in EGCs as highlighted by a significant and strong colocalization with GFAP (Fig. S1*D*) or S100β (Fig. S1*E*). Taken together, these results demonstrate that EphB2 is expressed by myenteric neurons in the soma and processes of enteric neurons, while Kalirin-7 is mainly found in EGCs and to some extent in neuronal processes.

### Dissecting EphB2 signaling in primary enteric neurons acutely exposed to ephrinB2

**Figure 2:**
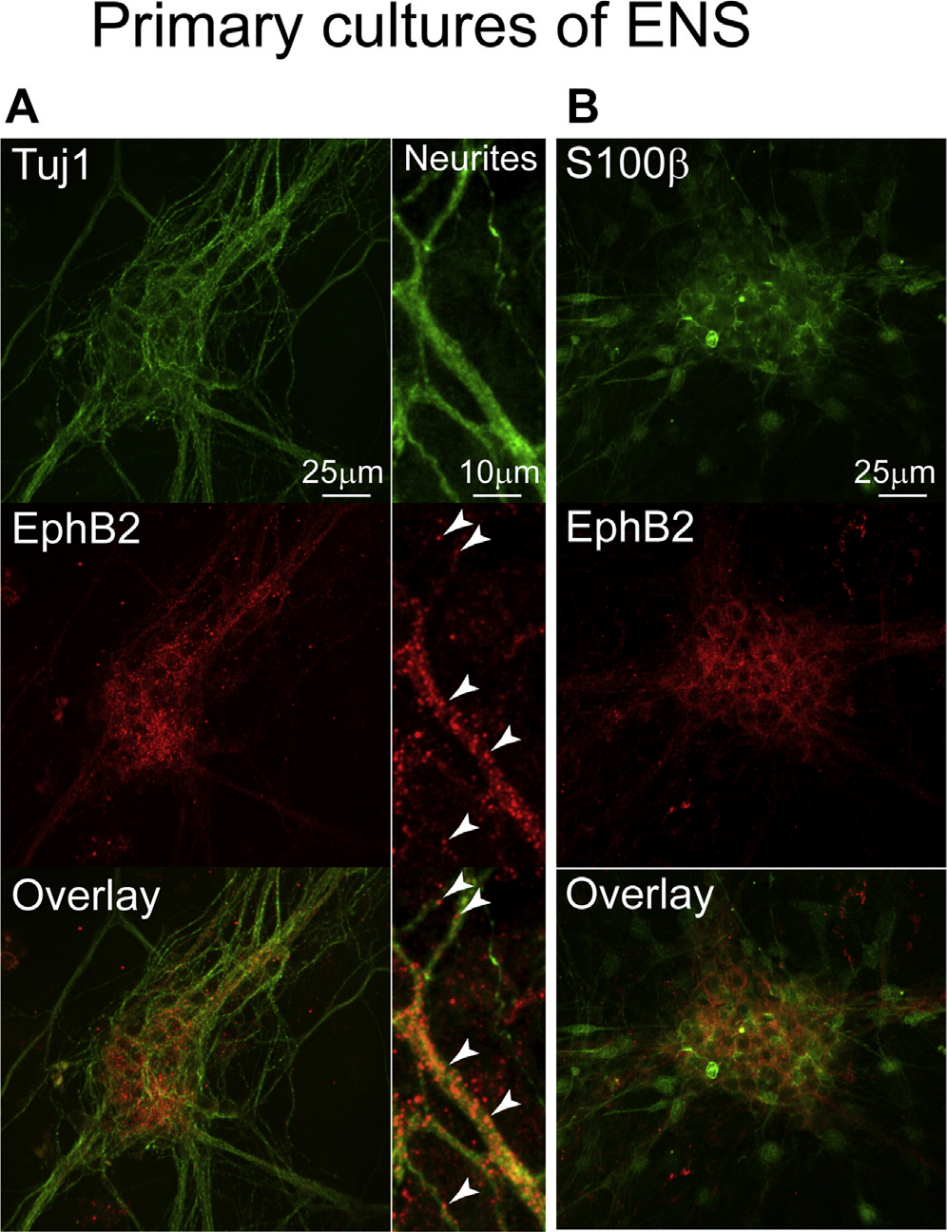
**EphB2 is expressed by enteric neurons in primary cultures of ENS.***A* and *B*, primary cultures of E15 rat intestines were grown on glass coverslips and fully matured into an enteric-like neuronal network with ganglia interconnected with neural fibers at 12 DIV. Cultures were then fixed and double-immunostained for EphB2 and tuj1 (*A*) or S100β (*B*). *Arrows* indicate punctae-like EphB2 localization within neuronal processes. Note that EphB2 is enriched within neuronal processes.

EphB2 signaling has been well characterized in primary hippocampal and cortical neurons (20), but much less in enteric neurons. Thus, we aimed to dissect EphB2 signaling in enteric neurons. To pursue this objective, we first examined whether primary cultures of enteric neurons (32, 33) are a suitable model system to study EphB2 signaling. We observed that EphB2 staining patterns are similar to that in colonic tissues. Indeed, EphB2 is mostly found in enteric neuronal processes, but not in EGCs, as shown by dual labeling with Tuj1 (Fig. 2*A*) and S100β (Fig. 2*B*) antibodies, respectively.

**Figure 3:**
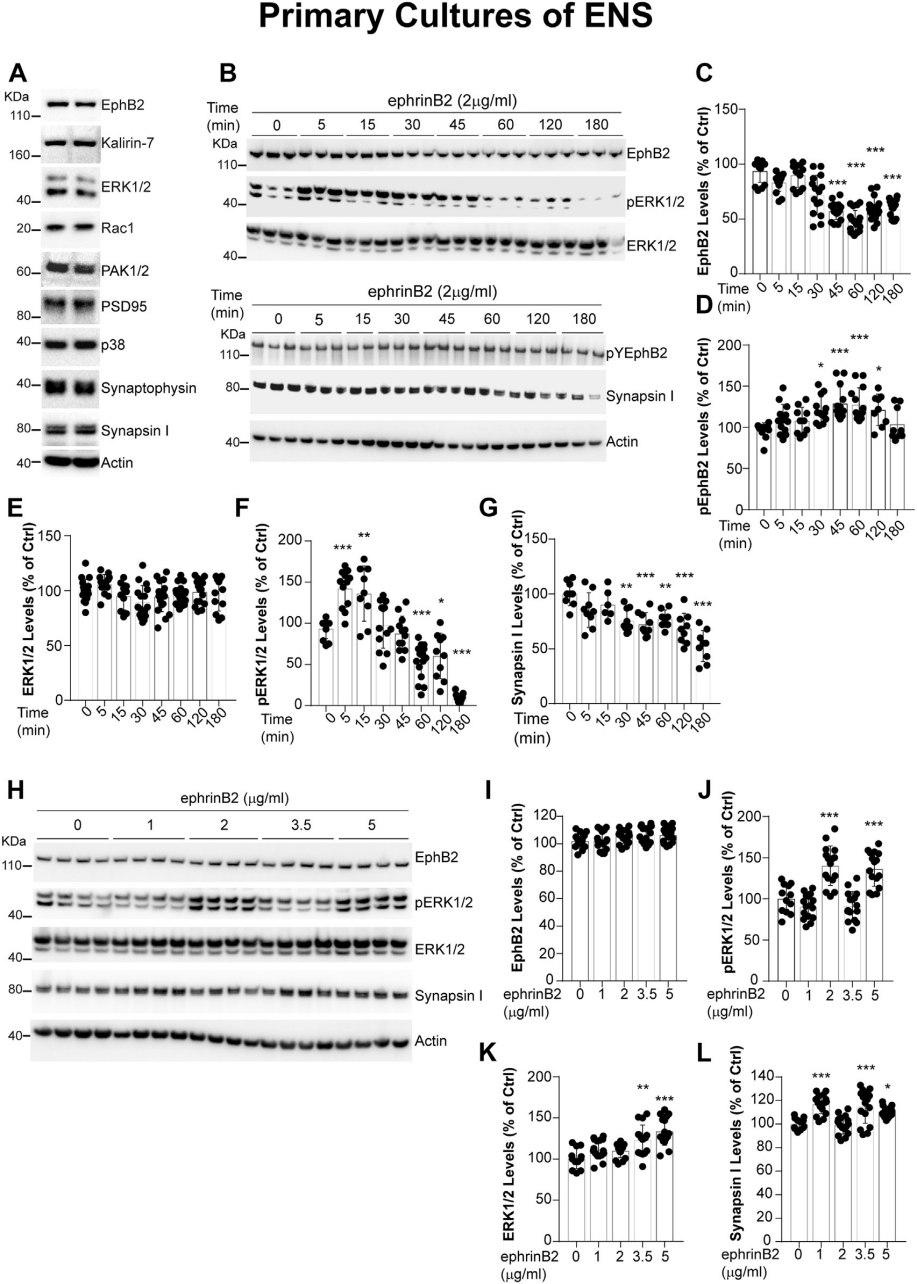
**EphB2 signaling in primary cultures of rat ENS.***A*, primary cultures of rat ENS were lysed at 12 DIV (days *in vitro*) and analyzed by western blot for detection of EphB2 downstream signaling molecules as indicated. The two lanes show repeats of two samples or two wells obtained from two distinct primary cultures of ENS. *B*, kinetic of ephrinB2-dependent activation of EphB2. Primary cultures of E15 rat intestines were treated at 12 DIV with clustered ephrinB2-Fc (ephrinB2) at 2 μg/ml for indicated time points. Cell lysates were then subjected to immunoprecipitation and/or direct western blot analysis for detection of full-length or active EphB2 and signaling/interacting molecules as indicated. *C*–*G*, quantitation of western blot signals shown in (*B*). Phosphoproteins pEphB2 and pERK1/2 were normalized to their respective total protein levels (n = 7–18 wells per condition from at least three independent cultures, one-way ANOVA followed by Tukey's post hoc test). ∗*p* ≤ 0.01, ∗∗*p* ≤ 0.001, ∗∗∗*p* ≤ 0.0001. *H*, dose response of ephrinB2-dependent activation of EphB2. Primary cultures of E15 rat intestines were treated at 12 DIV with clustered ephrinB2-Fc (ephrinB2) for 30 min with indicated concentrations of clustered ephrinB2. Cell lysates were then subjected to immunoprecipitation and/or direct western blot analysis for detection of full-length or active EphB2 and signaling/interacting molecules as indicated. *I*–*L*, quantitation of western blot signals shown in (*H*). (n = 12–18 wells per condition from at least three independent cultures, one-way ANOVA followed by Tukey's post hoc test). ∗*p* ≤ 0.01, ∗∗*p* ≤ 0.001, ∗∗∗*p* ≤ 0.0001.

We next used this neuronal model to dissect EphB2 signaling. First, we examined whether molecular mediators that have been associated to EphB2 in primary neuronal cultures from the CNS are expressed by enteric neurons as well. Interestingly, western blot analysis demonstrated that Kalirin-7 (25), ERK1/2 (34), Rac1 (27), PAK1 (35), PSD95 (36), p38 (37), synaptophysin (38), and synapsin I (39) were expressed in ENS cultures (Fig. 3*A*). Next, we sought to determine whether EphB2 is active in this culture model. We used a recombinant form of the ephrinB2, a natural ligand to EphB2, fused to a fragment crystalizable (Fc) moiety that can be clustered with an anti-Fc antibody to form a bioactive ephrinB2 multimer referred to as ephrinB2-Fc. Preclustered ephrinB2-Fc ligands can be used to trigger a variety of cellular responses depending on the experimental paradigm (40).

First, we examined the time-dependent capacity of ephrinB2 to activate EphB2 in primary enteric neurons. Neurons were treated with clustered ephrinB2-Fc ligand or control-Fc fragment for 5 min to 3 h at a concentration of 2 μg/ml. We found that EphB2 is activated/phosphorylated and downregulated between 30 and 45 min interval (Fig. 3, *B*–*D*). However, while total ERK1/2 levels were unchanged, we observed a phosphorylation of ERK1/2 as early as 5 min treatment of neurons with ephrinB2 (Fig. 3, *B*, *E*, and F). Interestingly, synapsin I levels were also reduced by 30 min with EphB2 activation (Fig. 3, *B* and *G*). These results suggest that ephrinB2 has a time-dependent effect on EphB2 and its downstream mediators in enteric neurons. Next, we conducted an ephrinB2 dose response. Neurons were treated for 30 min with indicated ephrinB2 concentrations. We observed no effect on EphB2 levels (Fig. 3, *H* and *I*). However, we observed changes in ERK1/2, phosphorylated ERK1/2, and synapsin I that are dependent of ephrinB2 dose (Fig. 3, *H*–*L*). Taken together, this data suggests that ephrinB2 activates EphB2 in enteric neurons with subsequent time- and dose-dependent changes on downstream mediators.

### Long-term activation by ephrinB2 reduces EphB2 expression in enteric neurons

**Figure 4:**
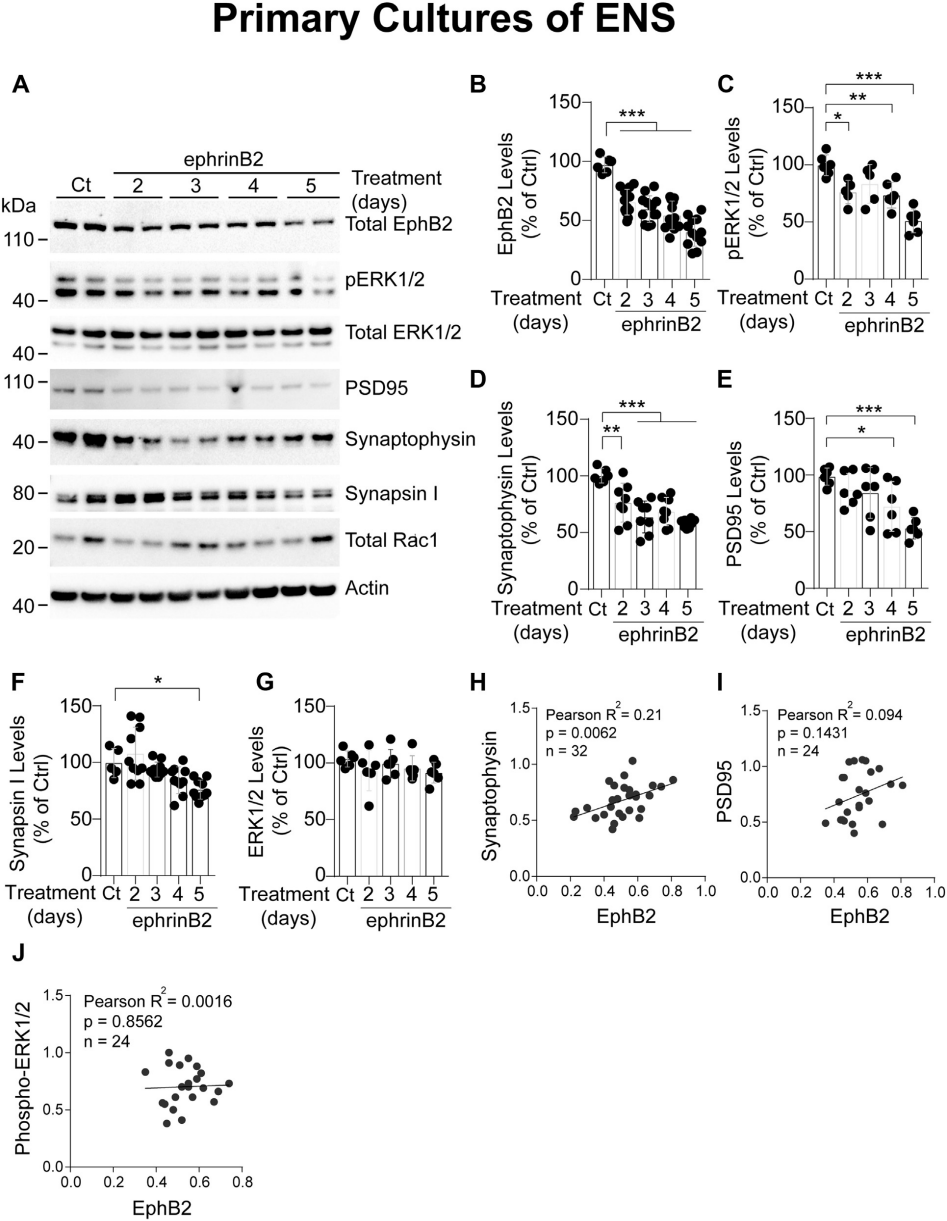
**EphB2 regulates the connectivity of enteric neurons.***A*, primary cultures of E15 rat intestines were treated at 12 DIV with control-Fc (Ct) or clustered ephrinB2-Fc (ephrinB2) for indicated times. Cell lysates were then subjected to immunoblotting assays. Note that levels of EphB2 and synaptic markers were reduced by ephrinB2 treatment. Actin was used as loading control. *B*–*G*, quantitation of western blot signals shown in (*A*). (n = 10–12 wells per condition from at least three independent cultures, one-way ANOVA followed by Tukey's post hoc test). ∗*p* ≤ 0.05, ∗∗*p* ≤ 0.01, ∗∗∗*p* ≤ 0.001. *H*–*J*, correlation analysis of EphB2 levels to synaptic markers in primary enteric neurons. Loss of synaptophysin (*H*, slope = 0.4883 ± 0.166, R^2^  = 0.224, n = 32, *p* = 0.0062), but not PSD95 (*I*, slope =  0.6202 ± 0.4084, R^2^ = 0.0949, n  =  24, *p* = 0.1431) or phosphorylated ERK (pERK) (*J*, slope = 0.07452 ± 0.406, R^2^  =  0.0017, n  = 22, *p*  = 0.8562) correlates with EphB2 depletion induced by a prolonged activation by ephrinB2 in ENS primary cultures. A positive trend indicates that synaptophysin concomitantly decreases with EphB2.

Next, we examined the impact of EphB2 activation on the connectivity of enteric neurons. To this end, we chronically exposed enteric neurons to clustered ephrinB2 or control for 2 to 5 consecutive days. Similar to an acute activation, we observed a sustained decrease in levels of total EphB2 with a concomitant loss of pERK1/2 (Fig. 4, *A* and *C*). To assess the impact on neuronal connectivity, we evaluated levels of various SAPs such as synapsin I and synaptophysin, which are abundantly present in presynaptic neuronal vesicles, and PSD95, the major scaffolding protein in the excitatory postsynaptic density. We observed that ephrinB2-induced depletion of EphB2 was accompanied with a significant decrease of synaptophysin (Fig. 4, *A* and *D*), PSD95 (Fig. 4, *A* and *E*), and to a lesser extent synapsin I (Fig. 4, *A* and *F*). In contrast, total ERK1/2 (Fig. 4, *A* and *G*) and total Rac1 (Fig. 4*A*) were unaffected. Correlation analysis by densitometry revealed that levels of synaptophysin (Fig. 4*H*), but not PSD95 (Fig. 4*I*), or pERK1/2 (Fig. 4*J*), were linearly correlated with EphB2 levels.

### Long-term activation of EphB2 by ephrinB2 reduces enteric neuronal connectivity

**Figure 5:**
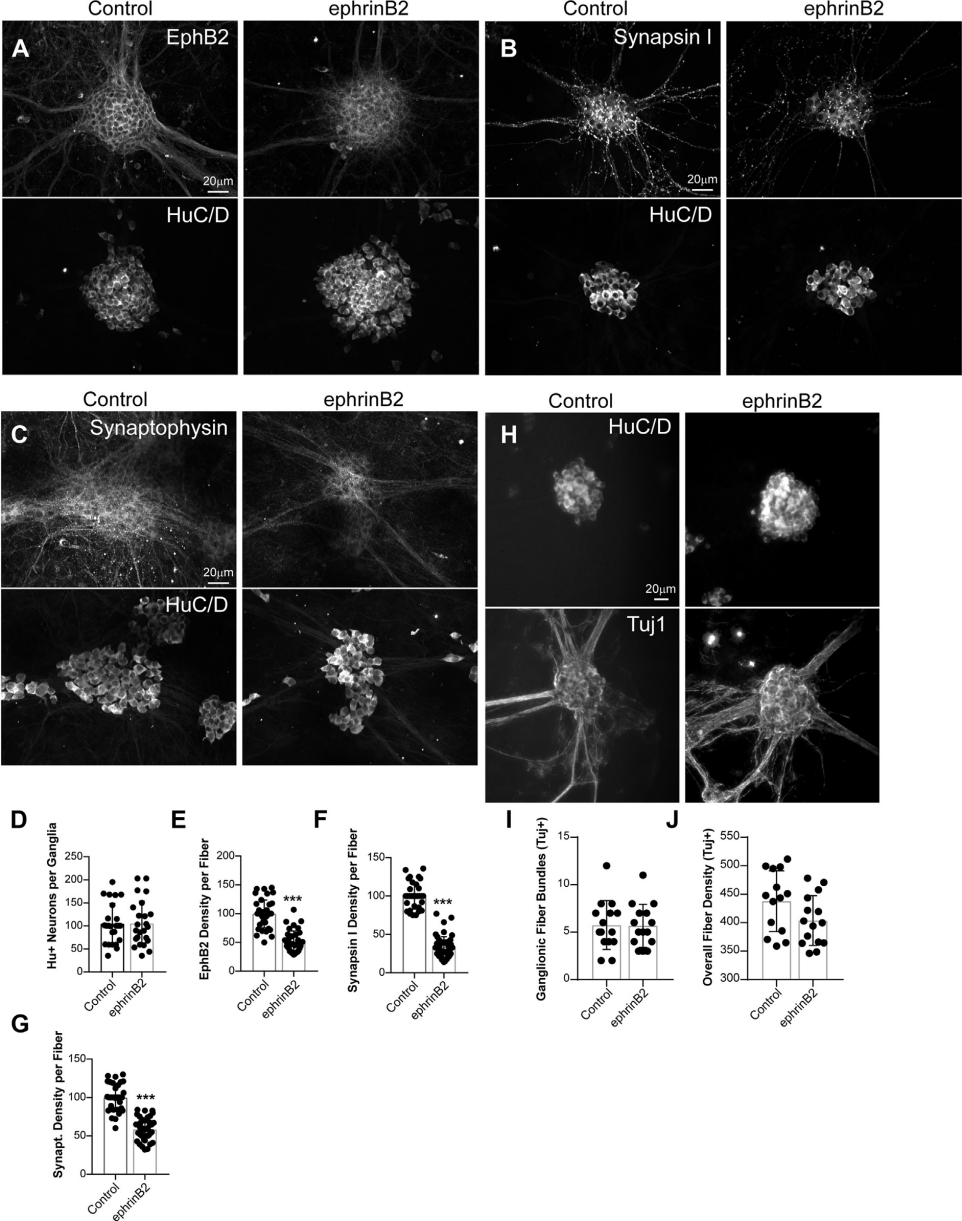
**Chronic activation with ephrinB2 reduces levels of EphB2 and synaptic-associated proteins in ENS cultures.***A*–*C*, primary cultures of E15 rat intestines were grown on glass coverslips and fully matured into an enteric-like neuronal network with ganglia interconnected with neural fibers at 12 DIV. Cultures were then treated with control-Fc (Control) or ephrinB2-Fc ligand (ephrinB2) for 5 consecutive days. Cultures were fixed and double-immunostained for EphB2 (*A*), synapsin I (*B*), synaptophysin (*C*) and pan-neuronal marker HuC/D (*A*–*C*). Images represent ganglia depicting neuronal cell bodies and processes. *D*–*F*, quantitations of images represented in (*A*–*C*) for HuC/D-positive neurons (*D*), and neuronal fiber density of EphB2 (*E*), synapsin I (*F*) or synaptophysin (*G*). Note that chronic exposure of neurons to ephrinB2 decreased EphB2 expression within neuronal processes without affecting overall neuronal density. Synapsin I and synaptophysin were reduced within neuronal processes. *H*, images represent ganglia depicting neuronal cell bodies (HuC/D staining) and neuronal processes (Tuj-1 staining). *I* and *J*, quantitations of images represented in (*H*) for the number of neuronal processes/ganglionic fiber bundles (*I*) and overall fiber density (*J*). (n = 10–15 ganglia per condition from at least three independent cultures, unpaired *t* test). ∗∗∗*p* ≤ 0.0001.

To further explore whether EphB2 activation affects neuronal connectivity, we exposed primary cultures of enteric neurons to ephrinB2 or control fragment for 5 consecutive days. First, we performed an immunohistochemical analysis to visualize synapses with antibodies that label synapse-specific components in pre- and/or postsynaptic compartments (41). We found that exposure to ephrinB2 reduced EphB2 (Fig. 5, *A* and *E*), with no change in overall neuronal density as assessed by staining of neurons with anti-HuC/D antibody (Fig. 5, *A* and *D*). Furthermore, we found that exposure to ephrinB2, but not control fragment, reduced the expression of synapsin I (Fig. 5, *B* and *F*) and synaptophysin (Fig. 5, *C* and *G*).

Next, we examined whether ephrinB2-dependent activation of EphB2 affects overall neuronal process morphology through Tuj-1 staining. We found no morphological difference in neurite growth and number in ephrinB2-treated relative to control condition as assessed by Tuj1 staining (Fig. 5, *H*–*J*). This suggests that depletion of EphB2, synapsin I, PSD95, or synaptophysin following treatment of neurons with ephrinB2 is more likely due to ephrinB2-specific effect on EphB2 and downstream regulatory mechanisms rather than a broader effect reducing overall neuronal process number, growth, and SAPs.

**Figure 6:**
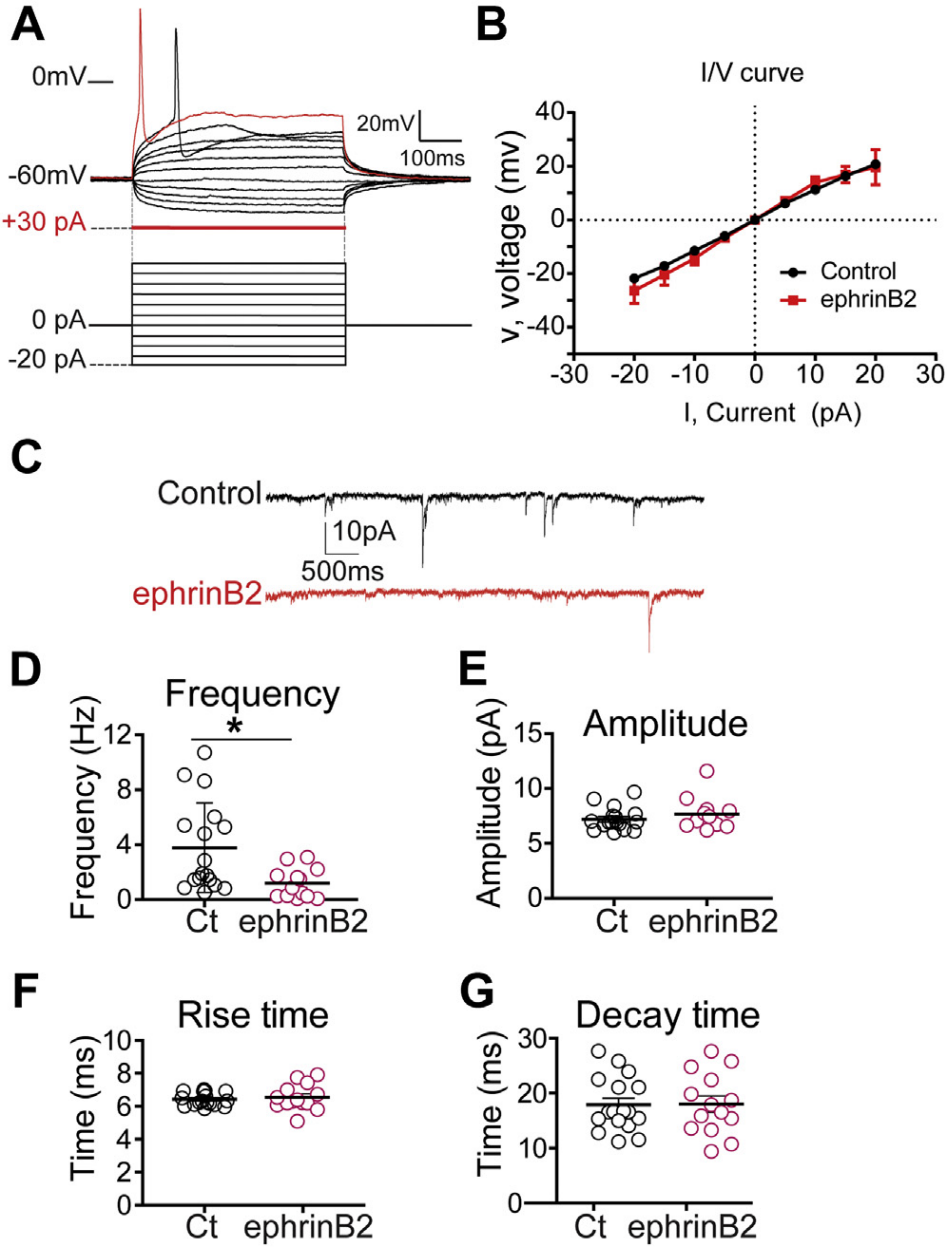
**EphB2 controls synaptic inputs in enteric neurons.***A*–*G*, primary cultures of ENS were grown for 12 DIV. Cultures where then stimulated with control (Ct) or ephrinB2 for 5 consecutive days. *A*, characteristic membrane properties and spiking pattern of primary enteric neurons obtained with a KCl-based intracellular solution. *Raw traces* show individual voltage responses to series of 500 ms current pulses from −20 pA to +30 pA with 5 pA steps (*black traces*) and to +30 pA above AP threshold (*red traces*). *B*, enteric neurons steady-state average I/V relationship from neurons recorded with a KCl-based internal solution (*red curve*: ephrinB2; *black curve*: control). Passive and active membrane properties of enteric neurons recorded in current-clamp configuration using a KCl-based intracellular solution are summarized in Table 1. *C*, representative traces of mPSCs recorded from enteric neurons stimulated with Control-Fc (Control) or ephrinB2-Fc (ephrinB2). *D*, quantification of mPSC frequency, mean_Control *versus* ephrinB2_: 3.781 ± 0.79 Hz *versus* 1.184 ± 0.3 Hz; *p* = 0.0136, n = 13–17 neurons per condition, Mann-Whitney test, Control/ephrinB2: U = 52. *E*, amplitude, mean_Control *versus* ephrinB2_: 7.195 ± 0.25 pA *versus* 7.647 ± 0.39 pA; *p* = 0.3003, Mann–Whitney test, Control/ephrinB2: U = 85. *F*, rise time, mean_Control *versus* ephrinB2_: 6.420 ± 0.1 ms *versus* 6.542 ± 0.21 ms, *p* = 0.7101, Mann–Whitney test, Control/ephrinB2: U = 109. *G*, decay time, mean_Control *versus* ephrinB2_: 17.88 ± 1.2 ms *versus* 18 ± 1.49 ms; *p* = 0.9844, Mann–Whitney test, Control/ephrinB2: U = 118. For all recordings, n = 13–17 neurons from at least five independent cultures, nonparametric Mann–Whitney test; ∗*p* < 0.05.

To examine whether chronic changes in neuronal connectivity markers induced by chronic exposure (5 days) to ephrinB2 translate into changes in electrophysiological properties of primary culture of enteric neurons, we performed whole-cell patch-clamp recordings by using a KCl-based intracellular solution. We observed no difference in active and passive membrane properties in ephrinB2 relative to control-treated neurons (Table 1 and Fig. 6, *A* and *B*). Next, we used a Cs-based intracellular solution to measure miniature postsynaptic currents (mPSCs). Interestingly, we observed that neurons exposed to ephrinB2 exhibited a 3-fold reduction of mPSC frequency relative to control condition (*i.e.*, neurons exposed to control-Fc fragment) (Fig. 6, *C* and D). However, neurons exposed to ephrinB2 displayed normal amplitude (Fig. 6*E*), rise time (Fig. 6*F*), and decay time (Fig. 6*G*). These results further suggest that EphB2 regulates neuronal connectivity/activity.

**Table 1:** Summary of passive and active membrane properties of enteric neurons recorded in current-clamp configuration using a KCl-based intracellular solution

	Action potential							
	No. of cells	Vrest, mV	Rin, Mohm	Rheobase, pA	Amplitude, mV	Rise time, ms	Decay time, ms	Half-width, ms
Control	16/8	−38.2 ± 2	1615 ± 238	28.8 ± 3.9	52.2 ± 2.9	4 ± 0.3	6.2 ± 1.1	4.9 ± 0.6
ephrinB2	16/8	−37.6 ± 1.4	1602 ± 217	25.4 ± 4.4	57 ± 3.5	3.4 ± 0.3	5.4 ± 0.8	4.3 ± 0.6

## Discussion

This study provides evidence that EphB2 receptor regulates enteric neuronal connectivity and identifies underlying molecular mechanisms. We show that EphB2 is expressed by myenteric neurons across intestinal segments of the rat. EphrinB2-dependent activation of EphB2 engages ERK1/2 signaling pathway in ENS primary cultures. Overtime, this leads to a downregulation of EphB2 signaling with a subsequent depletion of synaptic proteins. Furthermore, long-term activation of EphB2 significantly reduced mPSPs of enteric neurons without affecting action potentials or resting membrane properties.

The broad expression of EphB2 in the gut suggests that it has an important role in the small and large intestines. However, we observed that expression of EphB2 mRNA is not consistent with that of its protein, probably due to posttranscriptional regulations that affect the mRNAs before translated into protein (42, 43).

In addition, the present study showed EphB2 expression in neuronal processes and to a lesser extent in cell body within the myenteric plexus. Although EphB2 could not be detected in enteric glial cells by immunostainings, we cannot rule out the possibility that it is expressed by these cell types (44, 45).

A comparison of EphB2 expression in the ENS and the CNS reveals some similarities. Indeed, EphB2 is not restricted to a specific region and highly expressed by neurons relative to glial cells in the CNS (17, 18) and ENS (this study). Furthermore, EphB2 is localized at synaptic sites in the CNS (46) and in interganglionic connections and neuronal processes in the ENS. However, Kalirin-7, a well-described molecule that mediates EphB2 signaling in the CNS (25, 29, 47), is highly expressed by glial cells relative to neurons in the ENS.

Our study also revealed that activation of EphB2 by ephrinB2 led to its phosphorylation in enteric neurons in a similar fashion to cortical or hippocampal neurons (48, 49). The resulting autophosphorylation sites have been characterized in neuronal cells from the CNS (50), but remain unidentified in the ENS. Another mechanism by which EphB2 activity is regulated is that a sustained activation by ephrinB2 leads to EphB2 cleavage within the transmembrane region by matrix metalloproteinases and γ-secretase activities (51, 52, 53). Similar mechanisms of regulation could occur in the ENS to control the duration of EphB2 response to a stimulus. This hypothesis is supported by our observation that EphB2 depletion translated into reduced downstream signaling, thereby limiting an excessive response or to allow a fine-tuning formation of neuronal connections.

EphrinB2-dependent activation of EphB2 engaged ERK1/2 signaling pathways in the ENS (this study). This is consistent with a previous study (31). Besides ERK1/2, our study demonstrated the presence in the ENS of molecular mediators such as Rac1, PAK1, p38, and Kalirin-7. Interestingly, these same signaling molecules mediate EphB2 signaling in the CNS as well, suggesting that EphB2 fulfills similar functions in both systems. An acute (≤3 h) or chronic (≥48 h) activation by ephrinB2 decreased EphB2 expression, thereby leading to changes in levels of downstream signaling molecules. Dose- and time-dependent analysis showed that ephrinB2-dependant activation of EphB2 engages downstream signaling molecules within minutes while its downregulation, which is more sensitive to activation time rather than dose, occurs at a later time point (45 min to 1 h). This observation might reflect a mechanism by which EphB2 is regulated to maintain a fine-tuned control of neuronal signaling system, as discussed in the previous paragraph. In contrast, a study by Zhang *et al*. (31) did not report a reduction of EphB2 or downstream signaling molecules upon exposure to ephrinB2 ligand. Differences in experimental paradigms (primary cultures of ENS *versus* colonic LMMPs) and methodological details with respect to ephrinB2 concentrations and length of cell exposure to ephrinB2 could account for these conflicting observations.

We observed that depletion of EphB2 and key SAPs (*i.e.*, PSD95, synapsin I, and synaptophysin) is not accompanied by an overall reduction in number and growth of ganglionic fiber bundles. This observation suggests that ephrinB2 affects SAPs through a specific activation of EphB2, rather than a broad negative effect on neuronal morphology and network. Indeed, depletion of EphB2 subsequently decreased levels of SAPs in the ENS, suggesting a control of their synaptic availability as a stabilizing molecule through complex formation or indirectly through transcription factors (49). Additional studies are warranted to determine whether EphB2 colocalizes and directly or indirectly interacts with PSD95, synapsin I, or synaptophysin at enteric neuronal connections.

Lastly, EphB2 depletion in primary ENS culture drastically decreased the frequency, but not amplitude, of mPSC. This decrease in the PSC frequency may reflect a synaptic disorganization, which could be explained by: (1) presynaptic mechanisms involving a decrease in neuronal connectivity and a reduced probability of neurotransmitter release; (2) postsynaptic mechanisms with a complete reorganization of proteins assemblies forming PSDs (54, 55). These hypotheses are not mutually exclusive. Previous studies have shown that knockdown of EphB2 in cortical neurons by siRNA reduced the frequency of miniature EPSCs (mEPSCs) from cells, with no effect on mEPSC amplitude (56, 57). Furthermore, synaptic currents are significantly reduced in mice lacking EphB2 in dentate gyrus granule cells (18) with significant alterations of overall neuronal plasticity such as long-term potentiation (17). Altogether, our results strongly suggest that EphB2 has similar functions in the gut and brain, which is to regulate neuronal connectivity and activity by stabilization of synaptic structure and organization of neuronal circuits through control of actin network.

In conclusion, the present study establishes EphB2 as a regulator of neuronal connectivity and activity in the ENS. It also provides a structural basis for understanding the physiology of EphB2 in the gut and potential involvement in pathologies with alterations in enteric neuronal connectivity. It would be important to determine in future studies whether loss of EphB2 and subsequent decrease in neuronal connectivity in the gut might constitute early pathology hallmarks in neurological pathologies with intestinal comorbidities.

## Experimental procedures

### Rats

Pregnant female (9–12-week-old) Sprague Dawley rats were purchased from Janvier Labs and embryo (E15) were used for primary mixed cultures of ENS. For analysis of intestinal segments, 6-month-old rats were used. All housing and experimental procedures for pregnant rats were carried out in compliance with the local ethical review panel of Inserm (agreement E. 44011; Inserm) and in accordance with the Council Directive 2010/63EU of the European Parliament and the Council of September 22, 2010 on the protection of animals used for scientific purposes. Animals were housed in standard 42 × 26 × 15 cm plastic cages. Bedding consisted of kiln-dried aspen shavings (SAFE). Water and food were available (SAFE) ad libitum. Animals were housed at 19 to 22 °C, humidity 55%, and 12:12 h light: dark cycle.

### Primary mixed cultures of ENS

Primary culture of rat ENS was performed as previously described (32). Briefly, embryonic day 15 (E15) rat intestine was removed and finely diced in Hank's buffered salt solution and triturated mechanically using a scalpel. Tissue fragments were collected in Dulbecco's modified Eagle medium (DMEM)/F12 (1:1) medium (Life Technologies) containing 50 μg ml^−1^ streptomycin and 50 U ml^−1^ penicillin and incubated for 15 min at 37 °C in the same medium containing 0.25% trypsin (Invitrogen) and 10% fetal bovine serum (FBS) to inactivate trypsin. Samples were incubated for 10 min at 37 °C with 0.1% DNase I (Sigma). After trituration and centrifugation for 10 min at 50*g*, cells were plated in DMEM/F12 containing antibiotics and 10% FBS at a density of 2.4 × 10^5^ cells cm^−2^ on 24-well plates previously coated with 0.5% gelatin (Sigma) for 24 h. Medium was replaced with fresh DMEM/F12 without FBS but supplemented with 1% N-2 (Invitrogen). Half of the medium was replaced every 4 days, and primary cultures were maintained for 12 days.

### Clustering of recombinant ephrinB2

To activate EphB2 receptor in mixed enteric cultures or colonic tissues, we used ephrinB2-Fc chimera (R&D Systems), preclustered with human IgG(Fc) fragment (Jackson ImmunoResearch Laboratories) before the application. To precluster, ephrin-B2-Fc, which connects mouse EphB2 to the Fc portion of the human IgG1 *via* a polypeptide linker, was mixed with anti-human Fc antibody (1:2) and incubated on ice for 30 min at room temperature. Eleven DIV primary cultures of ENS were stimulated for indicated times with clustered ephrinB2 at a concentration of 0.5 μg/ml. The human IgG(Fc) fragment was subjected to the same treatment and used as control. Cultures were then fixed or not for biochemical analysis or used for electrophysiological recordings.

### Immunohistochemistry

#### Intestinal segments

Different segments of adult Sprague Dawley rats (6-month-old) GI tract were fixed in 0.1 M PBS containing 4% paraformaldehyde at room temperature for 3 h at 4 °C. Whole mounts of longitudinal muscle and myenteric plexus were obtained by microdissection and were first permeabilized with PBS containing 4% horse serum and 0.5% Triton X-100. Tissues were then incubated with primary antibodies listed in Table 2 for 12 h at room temperature. After several washes in PBS, tissues were incubated for 1 h at room temperature with the appropriate FITC-conjugated or Alexa 568-conjugated secondary antibodies diluted in PBS containing 1% horse serum. Tissues were washed with PBS and mounted with ProLong Gold Antifade Reagents with DAPI (Molecular Probes).

**Table 2:** List of primary antibodies used for immunohistochemistry on tissues

Antibody	Specie	Concentration	Company	Reference
EphB2	Goat	2 μg/ml	Thermo Fisher	PA547017
Kalirin-7	Rabbit	1 μg/ml	Millipore	07122
PGP9.5 (31A3)	Mouse	1 μg/ml	ThermoFisher	MA1-83428
S100β clone SH-B1	Mouse	2 μg/ml	ABNOVA	MAB12455
GFAP	Mouse	2 μg/ml	Sigma	93893
HuC/D	Rabbit	2 μg/ml	Invitrogen	PA579199

#### Primary cultures of ENS

Fourteen DIV primary cultures of enteric neurons were fixed in PBS containing 4% paraformaldehyde for 15 min after various treatments. Cells were permeabilized for 5 min at room temperature in 0.25% Triton-X-100 in PBS, washed twice with PBS, and incubated for 30 min at 37 °C in PBS containing 10% BSA. Neurons were incubated overnight at 4 °C with primary antibodies diluted in PBS containing 3% BSA and 0.02% azide. Antibodies used are listed in Table 3. After washing, cells were incubated for 90 min at room temperature with the appropriate FITC-conjugated or Alexa 568-conjugated secondary antibodies diluted in PBS containing 3% BSA and 0.02% azide. Cells were washed with PBS and mounted with ProLong Gold Antifade Reagent with DAPI (Molecular Probes).

**Table 3:** List of primary antibodies used for immunohistochemistry on ENS cultures

Antibody	Specie	Concentration	Company	Reference
EphB2	Rabbit	2 μg/ml	Abcam	ab150652
Synapsin-1(D12G5)	Rabbit	2 μg/ml	Cell signaling	5297
Synaptophysin (7H12)	Mouse	2 μg/ml	Cell signaling	12270
PSD95 (6G6-1C9)	Mouse	6.6 μg/ml	ThermoFisher	MA1-045
βIII-Tubulin SDL.3D10	Mouse	4 μg/ml	Sigma	T8660-0.2ML
PGP9.5 (31A3)	Mouse	1 μg/ml	ThermoFisher	MA1-83428
GFAP (SMI 26)	Mouse	2 μg/ml	Eurogentec	SMI-26R-500
GFAP (SMI 26)	Mouse	2 μg/ml	Biolegend	BLE837602
S100β clone SH-B1	Mouse	2 μg/ml	ABNOVA	MAB12455
Hu	Human		Gift from CHU Nantes	

### Imaging and data analysis

#### Imaging

Capture of longitudinal muscle and myenteric plexus images was performed with the confocal microscope Nikon A1 RSi (Nikon SAS) with 20× /0.75 or 60× /1.4 oil immersion objectives.

Images of ENS cultures were captured using the fluorescent microscope AxioZoom.V16 (Zeiss) associated with Zen 2012 software (Zeiss). ImageJ software (NIH) was used for analysis and quantifications. All of the error bars denote standard error of the mean.

### Analysis of images

#### Fiber density

Fiber density was examined on primary cultures of SNE plated in 12-wells at ∼900 × 10^3^ density/well on poly-l-lysine-coated coverslips and immunolabeled with TUJ1. The fluorescent intensity of TUJ1-immunoreactive (IR) fibers above a fixed threshold using an area free from fibers for background subtraction was used to estimate a fiber density. Fiber density represents the value of the fluorescent intensity after background subtraction. The optical density (OD) value of each fiber density was derived from values measured using a square area of constant volume placed at the junction of the fibers innervating a ganglion. The overall fiber density represents the sum of OD values of all fiber density departing/projecting from a ganglion. Each data point on the graph represents the OD of all fibers density from at least ten ganglia per well that was averaged by the number of ganglia counted. OD values of ganglionic fibers were compared with those in the control group. Fifteen wells from three independant experiments/cultures were counted per condition. EphB2, synapsin I, and synaptophysin density per fiber was obtained as outlined above except that a specific antibody was used to obtain OD values.

#### Ganglionic fiber bundles

This data represents the number of fibers departing/projecting from a ganglion and examined with TUJ1 staining. Values were obtained by visually/manually counting the number of outgoing fibers per ganglion. Each data point represents an averaged number of ganglionic fiber per ganglion. The total number of fiber bundles from at least ten ganglia was averaged per well. Fifteen wells from at least three independant cultures were analyzed per condition.

#### HU+ neurons

Number of neurons per ganglion was obtained by counting each HU+ neurons within a ganglion. HU+ neurons were visually/manually counted using ImageJ cell counter plugin. At least ten ganglia were counted per well and averaged to obtain a data point represented on the graph. In total, 20 to 25 wells were counted per condition from at least three independent cultures.

For all quantification, the experimenter analyzed the pictures blindly. Density was expressed as a percentage relative to the control (EphB2, synapsin I, synaptophysin)/as arbitrary unit (overall fiber density per ganglion)/or as raw counted number (number of fiber bundles and number of neurons).

### Western blots

#### Primary cultures of enteric nervous system

Enteric mixed cultures were scrapped into cold PBS containing protease cocktail inhibitor, pelleted, and resuspended in Laemmli buffer. Cell lysates (10 μg) were separated using the Invitrogen NuPage Novex Bis Tris MiniGels (4–12% bis Tris) with the Mes-SDS running buffer before electrophoretic transfer to nitrocellulose membranes with the iBlot2 Dry Blotting System (Life Technologies). For phosphorylated EphB2 detection, 100 μg of proteins was mixed in 400 μl of binding buffer (50 mM Tris, pH 7.5, 200 mM NaCl, 0.1% NP-40) and rotated overnight at 4 °C. Avidin-agarose beads (40 μl of 75% slurry; Pierce) and 1 μg/ml anti-EphB2 antibody were added, and the tubes were rotated at 4 °C for 2 h and spun at 13,000 rpm for 30 s. The supernatant was discarded. Beads were washed twice with 500 μl of PBS and resuspended in 30 μl of 2× loading buffer. Samples were boiled at 90 °C and loaded onto a NuPAGE 4 to 12% Bis-Tris gel for western blot analysis. Gels were transferred to nitrocellulose membranes and immunoblotted with an anti-phosphotyrosine antibody. For all western blots, proteins that are different in size (*e.g.*, Kalirin-7, EphB2, PAK1/2, pERK1/2, and total Rac1) are from the same gel (gel cut off at the appropriate molecular weights before probing with antibody). If proteins are at similar sizes (*e.g.*, pERK1/2 and ERK1/2), additional gels were run to complete analysis. For each gel, actin was used as an internal loading control. Membranes were blocked for 1 h at 25 °C in Tris-buffered Saline-Tween 0.1% (TBST) (150 mm NaCl, 15 mm Tris, 4.6 mm Tris base, Tween 0.1%, pH 7.4) containing 5% nonfat dry milk and incubated overnight at 4 °C with primary antibodies listed in Table 4. Bound antibodies were detected with a horseradish-peroxidase-conjugated anti-rabbit or anti-mouse antibody (Thermo Fisher Scientific; diluted 1:5000) and visualized by chemiluminescence (Clarity Western ECL Substrate, Bio-Rad) using a Gel-Doc imager and the Image Lab Software (Bio-Rad).

**Table 4:** List of primary antibodies used for Western blot on tissues and ENS cultures

Antibody	Specie	Concentration	Company	Reference
EphB2	Rabbit	1 μg/ml	Cell signaling	ab150652
EphB2	Mouse	1 μg/ml	Life technologies	371700
Synapsin-1 (D12G5)	Rabbit	1 μg/ml	Cell signaling	5297
Synaptophysin (7H12)	Mouse	1 μg/ml	Cell signaling	12270
PSD95 (6G6-1C9)	Mouse	1 μg/ml	ThermoFisher	MA1-045
Kalirin-7	Rabbit	1 μg/ml	Millipore	07122
Anti-phospho-tyrosine	Mouse	1 μg/ml	Sigma	05321
Rac 1	Mouse	1 μg/ml	Millipore	05389
Phospho-ERK1/1	Rabbit	1 μg/ml	Cell signaling	9102S
ERK1/2	Rabbit	1 μg/ml	Cell signaling	9102S
β-actin	Mouse	0.5 μg/ml	Sigma	T8660
PAK1	Rabbit	1 μg/ml	Cell signaling	2604S

#### Gastrointestinal tissues

GI samples from 6-month-old rats were homogenized in RIPA lysis buffer (50 mM Tris, 150 mM NaCl, 1% NP40, 0.5% sodium deoxycholate, 0.1% SDS, 1 mM EDTA, pH8.0) and protease inhibitors (Complete; Roche), pH 7.4, with a tissue homogenizer (Precellys 24, Bertin Technologies) and sonicated three times for 5 s. Samples were then centrifuged at 20,000*g* for 20 min to collect the supernatant. Equal amounts of lysate were boiled at 90 °C and loaded onto a NuPAGE 4 to 12% Bis-Tris gel for western blot analysis as described above.

### Quantitative polymerase chain reaction

RNA from gut tissues of rat was isolated using the Nucleo Spin RNA Triprep Kit or Clean up (Macherey-Nagel), respectively, according to the manufacturer's instructions. Potential genomic DNA contamination was removed by treatment with Turbo DNase (Ambion Inc), and RNA was quantified using an ND-1000 UV-Vis spectrophotometer (Nanodrop Technologies). cDNA was synthesized from 1 μg total RNA using the Super Script III Reverse Transcriptase System kit (Invitrogen) and diluted to a final concentration of 8 ng eq RNA/μl. qPCR was performed using StepOne Plus (Life Technologies) detection system with Fast SYBR Green (Life Technologies) master mix. The PCR signal was normalized against S6 as reference gene to control for variability in the amount and quality of the RNA. The following primers were used to evaluate mEphB2 transcript levels: forward 5′ ACCTCAGTTCGCCTCTGTGAA 3′ and reverse 5′ GGCTCACCTGGTGCATGAT 3′.

### Electrophysiology

Enteric mixed cultures (SNE) cells were grown for 11 days on glass coverslips and incubated with the control-Fc fragment or ephrinB2-Fc for 2 days. Glass coverslips were then transferred into a recording chamber and continuously perfused with Ringer's saline buffer containing 140 mM NaCl, 4 mM KCl, 2.5 mM CaCl_2_, 1 mM MgCl_2_, 10 mM HEPES, 11 mM glucose, buffered to pH 7.4, at 22 to 24 °C (room temperature). Osmolarity was adjusted to 310 mOsm. Recording pipettes (5–7 MΩ resistance) were filled with two different internal solutions: (1) Current clamp recordings (active and passive membrane properties): 110 mM KCl, 30 mM kF, 1 mM MgCl2, 1 mM CaCl2, 10 mM HEPES, 2 mM EGTA, and 0.4 mM NaGTP and titrated to pH 7.4 with KOH. Osmolarity was adjusted to 300 mOsm. (2) Voltage clamp recordings (mPSCs recordings): recording pipettes (5–7 MΩ resistance) were filled with 135 mM CsCl, 0.3 mM EGTA, 10 mM HEPES, 4 mM MgATP, 0.3 mM NaGTP and titrated to pH 7.2 with CsOH. Osmolarity was adjusted to 300 mOsm. Cells were continuously perfused with extracellular solution, at a rate of 2 ml/min. Primary cultures of ENS were visualized under an Olympus BX51WI microscope (Olympus), with a 4×/0.13 objective for the placement of the stimulating electrode and a 40×/0.80 water-immersion objective for the localization of cells for whole-cell recordings. Voltage-clamp recordings were filtered at 5 kHz and sampled at 10 kHz, with the Patchmaster program (HEKA Elektronik). The series resistance was compensated at 75 to 80%. Spontaneous currents were measured using a Cesium-high-chloride-based intracellular solution adapted from Osorio *et al*. (58). Cells within a visible ganglion were patch-clamped. Using an internal solution with KCl in current-clamp configuration, we applied a series of 500 ms current pulses from −20 pA to +60 pA. The I/V relationship as well as active membrane properties were then measured using Fitmaster (HEKA Elektronik). Prior to recording mPSCs by using an internal solution with CsCl, neurons were identified by applying a series of 500 ms current pulses from −20 pA to +60 pA. Only cells with APs were used for mPSCs recordings (active properties could not be measured by using a Cs-based solution). Neurons were then voltage clamped at −60 mV and mPSCs were recorded and analyzed during 250 s recording segments. The spontaneous postsynaptic currents (sPSCs) were identified by using a semiautomated amplitude threshold-based detection software (Mini Analysis 6.0.7 Program, Synaptosoft).

### Statistical analysis

Investigators who obtained data were blinded to the treatment of cell cultures. Sample sizes were chosen on the basis of pilot experiments and our experience with similar approaches. Statistical analyses were performed with GraphPad Prism Data analysis and statistics were performed using GraphPad Prism 7. Group comparisons were made by one-way ANOVA followed by Tukey's or Mann–Whitney post-hoc test. Linear regression analysis was performed using the least-squares method. All *p* values < 0.05 were considered significant. Summary data are presented as mean ± standard deviation (SD).

## Data availability

All data are contained within this manuscript.

## Supporting information

This article contains supporting information.

## Conflict of interest

The authors declare that they have no conflicts of interest with the contents of this article.
