# Supplementary material for: The ephrin receptor EphB2 regulates the connectivity and activity of enteric neurons
Source: J Biol Chem. 2021 Oct 11;297(5):101300. doi: 10.1016/j.jbc.2021.101300 (PMC8569587; doi:10.1016/j.jbc.2021.101300)
Supplement: Figure S1 [file mmc1.pdf]

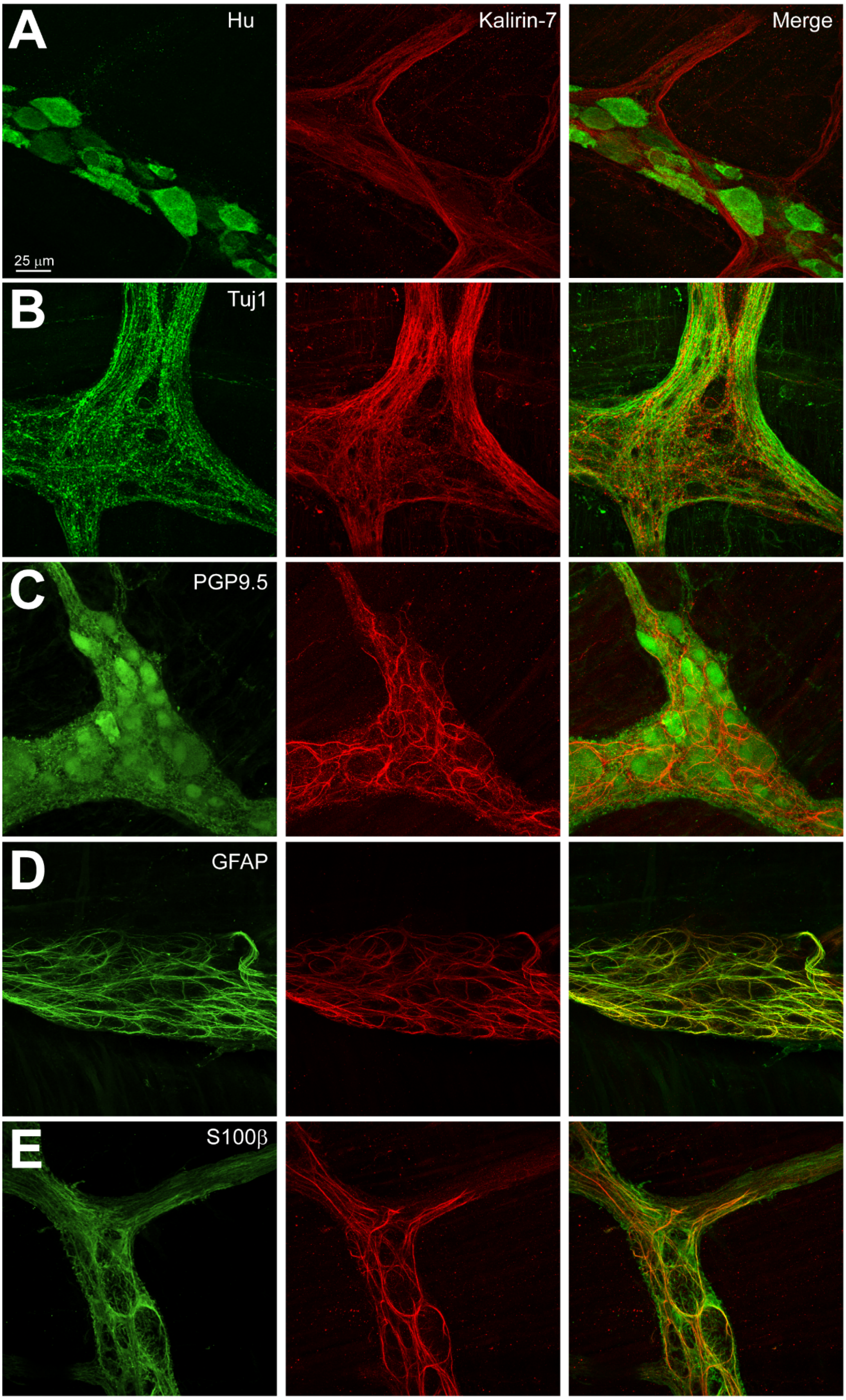

Figure S1

Figure S1 legend. Kalirin-7 is enriched in enteric glial cells. **A**-**E**, The myenteric plexus of distal colon from an adult rat (6-month-old) was double-immunostained for Kalirin-7 and HuC/D (**A**), Tuj1 (**B**), or PGP9.5 (**C**). Images show no evident colocalization of Kalirin-7 with neuronal somata. Double-immunostaining for Kalirin-7 and GFAP (**D**), or S100β (**E**) in a whole-mount preparation of myenteric plexus from distal colon of adult rat (6-month-old). Images show a very strong colocalization of Kalirin-7 with enteric glial cells.
